# Evidence for a magma reservoir beneath the Taipei metropolis of Taiwan from both S-wave shadows and P-wave delays

**DOI:** 10.1038/srep39500

**Published:** 2016-12-23

**Authors:** Cheng-Horng Lin

**Affiliations:** 1 Institute of Earth Sciences, Academia Sinica, Taipei, Taiwan; 2 Taiwan Volcano Observatory at Tatun, Taipei, Taiwan; 3 Dept. of Geosciences, National Taiwan University, Taipei, Taiwan; 4 National Center for Research on Earthquake Engineering, National Applied Research Laboratories, Taipei, Taiwan

## Abstract

There are more than 7 million people living near the Tatun volcano group in northern Taiwan. For the safety of the Taipei metropolis, in particular, it has been debated for decades whether or not these volcanoes are active. Here I show evidence of a deep magma reservoir beneath the Taipei metropolis from both S-wave shadows and P-wave delays. The reservoir is probably composed of either a thin magma layer overlay or many molten sills within thick partially molten rocks. Assuming that 40% of the reservoir is partially molten, its total volume could be approximately 350 km^3^. The exact location and geometry of the magma reservoir will be obtained after dense seismic arrays are deployed in 2017–2020.

The Tatun volcano group (TVG) is located near the administrated border between two large cities (Taipei, the capital of Taiwan, and New Taipei) in northern Taiwan (Fig. 1). The TVG is composed of more than 20 volcanoes^1^, with the major eruption occurred around 0.4 Ma^2^. Among them, Mt. Chihsin has the highest summit, with an elevation of 1,120 meters, and may be the youngest volcano^3^. Over 7 million residents, one-third the population of Taiwan, live within a 30 km radius of Mt. Chihsin. Mt. Chihsin itself is a mere 15 km away from the 508 m tall Taipei 101 super skyscraper landmark in downtown Taipei^4^. Thus, the TVG may have a significant impact on the Taipei metropolis if it is active again in the future. Of note, there are also two nuclear power plants operating in the vicinity of the TVG along the northern coast of Taiwan (Fig. 1).

It has been debated for decades whether the TVG contains active volcanoes. The results of an early study on lava dating suggested that the last volcanism at the TVG started at approximately 1.5 Ma and stopped around 0.1–0.2 Ma^5^,^6^,^7^. Thus, the TVG are often considered to be extinct volcanoes, according to the general identification of active volcanoes^8^. However, some recent analyses from geological, geochemical, geophysical, and seismic observations indicate the activity associated with volcanism is still significant at the TVG. The helium isotope ratio was found at ranges between 4.0 and 6.7, strongly implying that some mantle material might still be ascending into the shallow crust or surface^9^. The crustal resistivity measured by audio-magnetotellurics showed clear hydrothermal systems beneath the TVG^10^. The repeated leveling surveys indicated some significant crust deformation nearby the strong fumarole in the past several years^11^. Clustering micro-earthquakes^12^,^13^ and typical volcanic earthquakes such as tornillos and monochromatic event^14^ have been repeatedly identified in the TVG. The last eruption at the TVG from both ash dating^3^ and petrogenetic processes of effusive eruption^15^ may have been less than 10,000 years ago. These observations strongly suggest that the TVG may still be active; however, no direct evidence has previously shown any magma reservoir beneath the TVG in northern Taiwan.

## Geological Background

**Figure 1:**
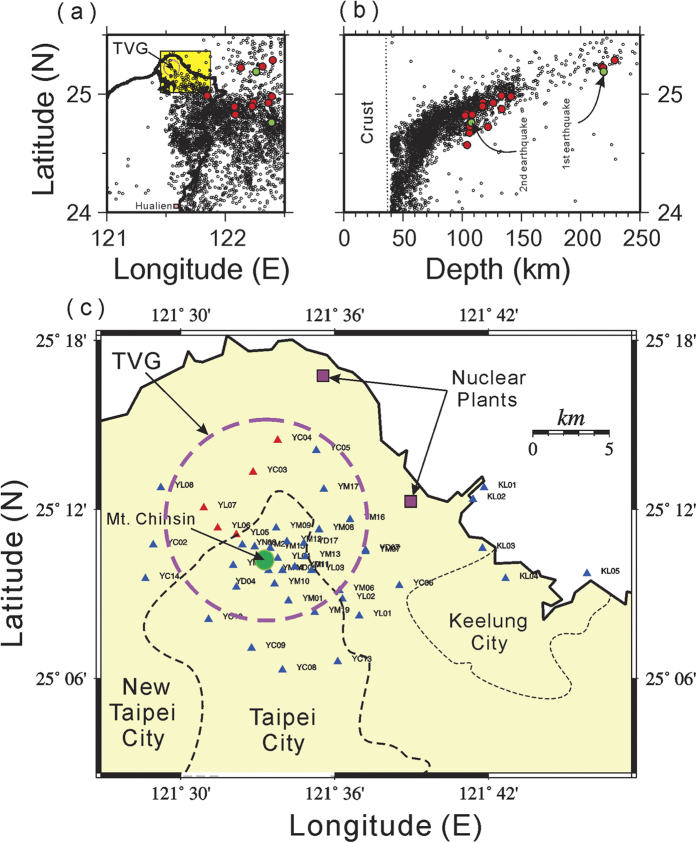
Locations of the TVG (big circle), background seismicity from 1994 to 2015 (small circles) and larger earthquakes in 2015 (red and green circles), major cities and seismic stations (triangles) in northern Taiwan. Background earthquakes (M > 3; depth > 40 km) and deeper earthquakes (M > 4; depth > 100 km) in 2015 projected on the map view (**a**) and the depth profile for showing the subduction zone (**b**). The two deep earthquakes analyzed in detail are marked by green circles. (**c**) Map showing seismic stations (triangles) in and around the TVG (big circle in red), two nuclear power plants (squares) and Mt. Chihsin (green circle) in and around the Taipei metropolis (grey box in (**a**)). The seismic station marked in blue and red show the seismogram recorded with and without clear S-waves, respectively. All figures were plotted with GMT v4.5.2 (gmt.soest.hawaii.edu).

From the tectonic point of view, the TVG is located at the western end of the subduction system in the northern Taiwan area (Fig. 1), where the Philippine Sea plate (PSP) is subducting northward beneath the Eurasian plate. All deeper earthquakes (depth > 40 km) occur in a limited area east of 121.5°E. The subduction slab of the PSP plate, shown by seismicity, starts from the Hualien area of eastern Taiwan (approximately 24°N) and gradually increases its depth down to more than 250 km beneath northeastern Taiwan (Fig. 1b). Although the TVG is located on the overlying plate of the subduction system, geochemical characteristics of varying from low-K to calc-alkaline and then shoshonitic compositions in the Northern Taiwan Volcanic Zone indicate that it might not be part of the typical volcanic arc induced directly by the subduction process^16^. Instead, the TVG volcanism might be the result of some degree of melting within an ascending region of asthenosphere mantle, due to the extensional collapse of the northern Taiwan mountain belt^17^.

## Seismic Network

In order to improve the capacity to detect the TVG volcanism, the seismic network at the TVG has been undergoing significant upgrades by the Taiwan Volcano Observatory at Tatun (TVO) since 2014. The seismic network started with only five short-period seismic stations near Mt. Chihsin in 2003^12^, and gradually expanded to 18 stations when the TVO was established in 2011^13^. In 2014, the total number of seismic stations dramatically increased from 18 to 40, covering an area of 15 km × 25 km. The station density with a spacing of approximately 1 km is relatively high near Mt. Chihsin, where volcanic earthquakes cluster^12^,^13^,^14^, but it is sparse over the area surrounding the TVG. Additionally, a few seismic stations (KL01-06) along the northern coast were added to detect possible volcanic seismicity from a submarine volcano just offshore from Keelung harbor. All 40 seismic stations have been upgraded from short-period to broadband seismic sensors (Guralp CMG-6TD) to allow the detection of a variety of volcanic earthquakes and tremors^13^,^14^,^18^,^19^,^20^.

## Results

### S-wave shadows

**Figure 2:**
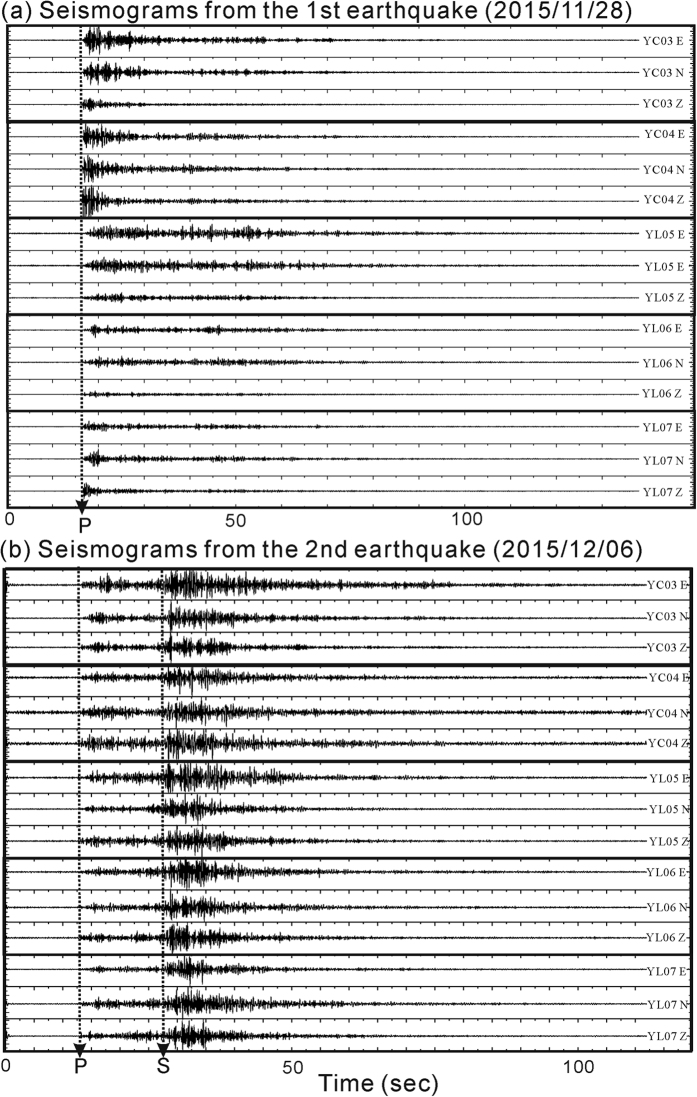
Comparison of three component seismograms between (**a**) attenuated and (**b**) clear S-waves generated by two representative earthquakes (No. 1 and 2 in Table S1) and recorded at Stations YC03-04 and YL05-07. This figure was plotted with SAC (version 101.5; URL: ds.iris.edu).

**Figure 3:**
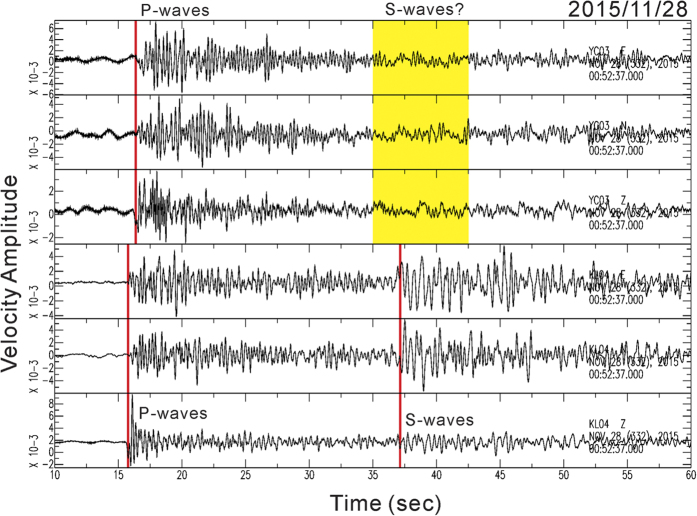
Three component seismograms recorded at two seismic stations (KL04 and YC03) showing the difference between attenuated (upper) and clear (lower) S-waves from the earthquake occurred on November 28, 2015. This figure was also plotted with SAC (version 101.5; URL: ds.iris.edu).

To examine the seismic waves propagating into a potential magma reservoir beneath the TVG, seismic data generated by deeper earthquakes (depth > 100 km) in 2015 were collected in accordance with the earthquake catalog provided by the Central Weather Bureau in Taiwan. In total, there were 20 earthquakes with the magnitudes greater than 4. Among them, two representative earthquakes (No. 1 and 2 in Table S1) provided information valuable for identifying the magma reservoir beneath northern Taiwan. The first representative earthquake had a local magnitude (M_L_) of 5.15 and was located within the subduction slab (122.261°E, 25.188°N). It occurred on November 28, 2015. Since this earthquake was located at a depth of 219 km beneath northern Taiwan, the incidence angles of the seismic waves at the seismic stations in the TVG were very close to vertical (70–80°). Thus, it was not surprising to see that most seismic stations recorded sharp arrivals of both P- and S-waves, as the major ray-path of the seismic waves propagated through the relatively homogeneous mantle. However, careful examination of three component seismograms recorded at the dense seismic network shows the shadow (attenuation) of S-waves at some neighboring seismic stations, such as YC03-04 and YL05-07, in the northwestern part of the TVG (Fig. 2a). Seismograms with and without S-waves could be unambiguously distinguished; particularly from the horizontal components (E-W and N-S) since the particle motion of S-waves was nearly horizontal (i.e., Fig. 3). Similar results observed from the 1^st^ representative earthquake have been consistently identified from another 3 deeper earthquakes (No. 3, 4 and 5 in Table S1) nearby the 1^st^ earthquake at the depths below 200 km (Fig. 1b). The S-wave attenuation from those 4 deeper earthquakes was clearly recorded at some neighboring seismic stations (Fig. S1).

### P-wave delays

**Figure 4:**
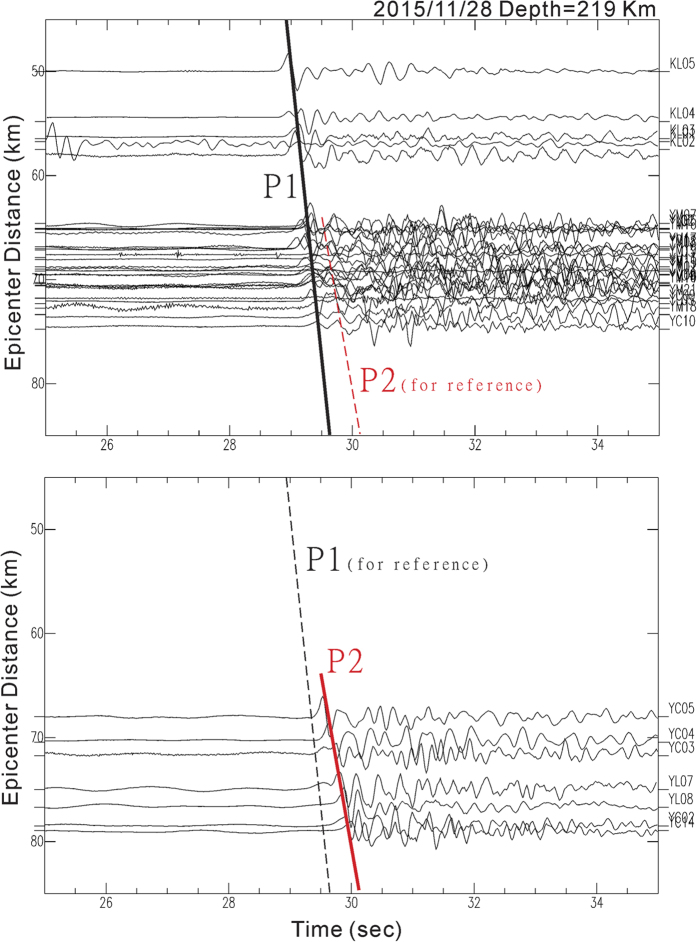
Plots of vertical seismograms with epicenter distances for showing the delayed P-waves (P2) in the lower panel in relation to the first P-waves (P1) in the upper panel recorded at seismic stations and generated by the earthquake (M5.15) on November 28, 2015. The thick lines are fitted by the arrivals, while the dashed lines are for reference. This figure was also plotted by SAC (version 101.5; URL: ds.iris.edu).

In addition to the S-wave shadows observed at some seismic stations, examination of P-waves recorded as the vertical component identified significant P-wave delays (Fig. 4). There were two groups of P-wave arrivals generated by the first representative earthquake (No. 1 in Table S1). The early group of P-waves recorded at most seismic stations at the TVG was approximately aligned with one straight line (P1), but the delayed group of P-waves recorded at particular stations such as YC02-05, YC14, and YL06-08 was roughly aligned with the other straight line (P2). Although there were still some slight differences (<0.1 s) between the P-wave arrivals at seismic stations and the straight lines due to the site conditions and elevation variations, on average, P2 was about approximately 0.4 s later than P1. It is also interesting to note that the P-wave delays were recorded not only at the seismic stations that showed S-wave shadows, but also at surrounding seismic stations (Fig. 1c). Similar to S-wave attenuation, the P-wave delay was also identified not only from the first representative earthquake but also another 3 deeper earthquakes (Fig. S2). The P-wave delays from 4 earthquakes were, respectively, plotted by contours (Fig. S3). Consistent results show P-wave delays ranging between 0.3 and 0.5 s were clearly clustering at seismic stations (black triangles) in the NW portion of the TVG.

### Deep magma reservoir

Both the S-wave shadows and P-wave delays identified from the first representative earthquake (No. 1 in Table S1) as well as another 3 deeper events (No. 3, 4 and 5 in Table S1) at depth greater than 200 km were difficult to find in the seismic data generated by other earthquakes at depths between 100 km and 150 km. Among them, for instance, the second representative earthquake (M_L_ = 4.32) was located within the subduction slab (122.393°E, 24.755°N and 108 km in depth) occurring on December 6, 2015 (No. 2 in Table S1). The three component seismograms recorded at Stations YC03-04 and YL05-07 showed that the S-wave arrivals were attenuated from the first representative earthquake, but easily identified during the second representative earthquake (Fig. 2b). Such a significant difference indicates the molten magma was neither just beneath the seismic station nor in the shallow crust because incidence angles of seismic waves at the TVG were nearly vertical for both earthquakes. Instead, the molten portion might exist in either the lower crust or the upper mantle along the ray-paths from the first earthquake to particular seismic stations.

### Ray-path and Travel-time estimation

**Figure 5:**
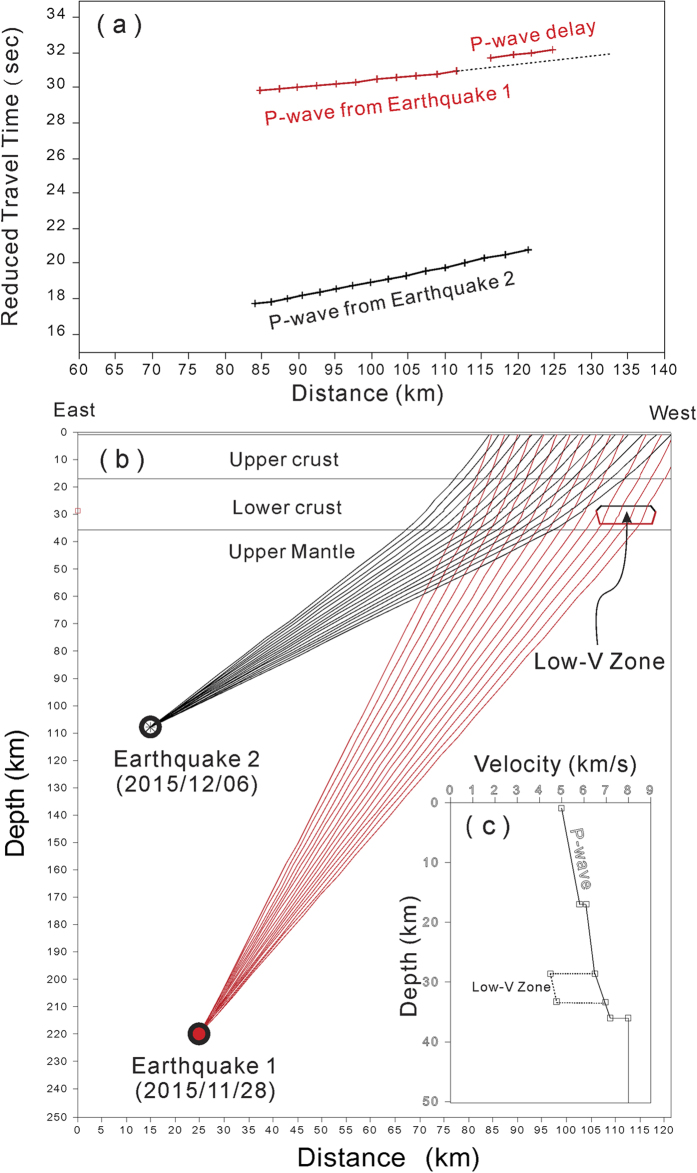
Simulation of (**a**) the calculated travel-times and (**b**) ray-paths generated by the two deep earthquakes (No. 1 and 2 in Table S1). A given 1-D velocity model with a low-velocity zone in the lower crust is also shown on the Fig. 5b.

In order to know the exact ray-paths and travel-times generated by the two representative earthquakes (No. 1 and 2 in Table S1) and recorded at the seismic array in the TVG, a synthetic calculation of MacRay^21^ is presented here. By using a simplified 1-D model of the crust overlying the upper mantle with a low-velocity zone (~40% less than the surrounding rocks) in the lower crust, the ray-paths and travel-times generated by two representative earthquakes and recorded at seismic stations at the TVG have been calculated along the E-W depth profile (Fig. 5). The incidence angles recorded at the TVG were ranging from approximately 70° to 80° for the first earthquake and from 50° to 60° for the second earthquake. The results show that most of the ray-paths propagated through the upper crust beneath the seismic array were overlaid on each other from two earthquakes. The separation of ray-paths generated by the two earthquakes is found in the upper mantle, as well as in a small portion of the lower crust on the westernmost part of the depth profile. The travel-times generated by the first earthquake at depth of 219 km show P-wave delay (~0.4 sec) at the right-hand side of Fig. 5b due to the low-velocity zone in the lower crust. The calculated results are consistent with the observations shown at Fig. 4, even though the exact location, size and velocity are not well constrained by the observations yet.

## Discussion

In order to exclude the possibility of S-wave shadow caused by the source radiation pattern effect, the focal mechanism of the first earthquake was determined by the 1^st^ motion P-waves recorded at seismic stations in the TVG as well as the Taiwan area (Fig. S4). Although the focal mechanism was not well constrained due to poor station coverage, the projection of the 1^st^ motions of the P-waves recorded at seismic stations in the TVG were limited within a small zone of the dilatational quarter in the focal mechanism. Also the 1^st^ motion of P-waves observed at those seismic stations were not far from one of the possible fault planes. Thus, the S-wave shadow observed at a few of neighboring seismic stations in the TVG couldn’t be caused by the source radiation pattern.

**Figure 6:**
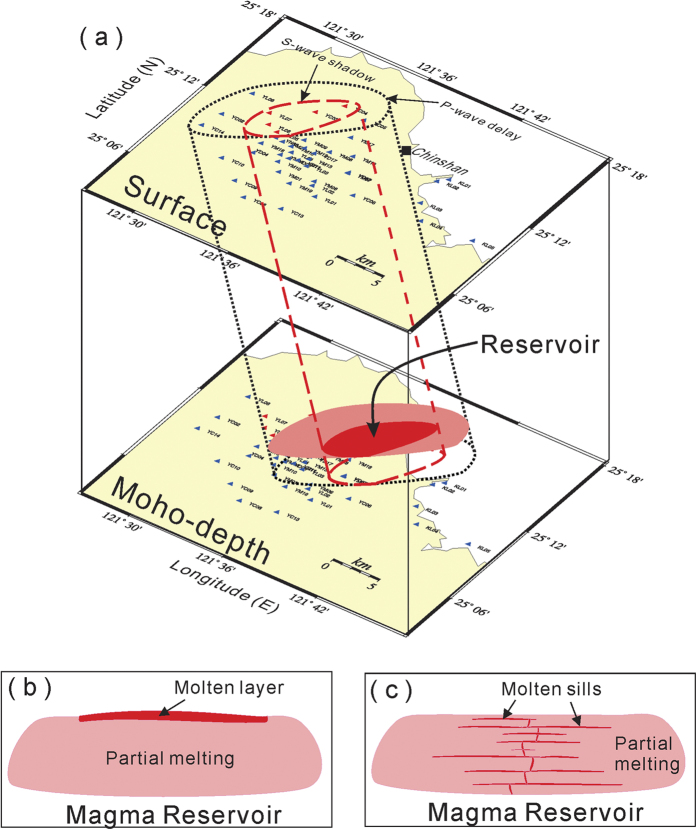
(**a**) Location projection of seismic stations (triangles), S-wave shadows (broken lines in red), and P-wave delays (dotted lines) on the surface down to the Moho depth based on the ray-paths from the deep earthquakes beneath northern Taiwan. Schematic plots for a magma reservoir composed of either (**b**) a thin molten overlay (red) or (**c**) many molten sills (red) within thick partially molten rocks (pink). All figures were plotted with GMT v4.5.2 (gmt.soest.hawaii.edu).

S-wave shadows (attenuation), like the early discovery of the liquid outer core of the earth^22^, provide evidence for the existence of a partially molten magma reservoir beneath northern Taiwan. The S-wave shadows recorded at stations YC03-04 and YL05-07 within an approximate area of 3 km × 8 km indicate molten magma exists somewhere along the ray-paths generated from the first earthquake (Fig. 6a). The S-wave shadows are hardly caused by any strong scattering from the Moho-discontinuity or complex structures in that the S-wave shadows are different from the coda waves, which often show seismic amplitudes decrease with time. The possibility of molten magma in the upper crust might be ruled out from the fact that no S-wave shadow was recorded from any seismic stations during the second earthquake. On the other hand, the velocity anomalies might not be located in the upper mantle because an extremely low velocity is required for causing P-wave delay of ~0.4 s within a small size of velocity anomalies limited by the area of the seismic stations that have P-wave delays. The extremely low velocity might not be reasonable in the partial melting of the upper mantle. Besides, P-wave delay of ~0.4 s recorded at seismic stations within a short distance (<10 km) in the Tatun volcano group of Taiwan is difficultly caused by the complex Moho-depth variation unless the seismic stations are just across the major plate boundary. Although there is currently not enough information to identify the exact depth of the magma reservoir, a reasonable estimation of its depth might be at the lower crust, such as the deeper reservoir beneath Yellowstone National Park in the USA^23^. Thus, the projection of the S-wave shadows down to the Moho depth, based on the calculated ray-paths, suggests the possible location of magma reservoir shifted approximately 10 km eastward at the lower crust, roughly beneath the Chinshan area (Fig. 6a).

In addition to the S-wave shadows, the existence of a magma reservoir can be strongly confirmed by the P-wave velocity delay at particular seismic stations at the TVG. In fact, the P-wave delay was detected not only at the seismic stations where S-waves attenuated, but also at surrounding stations in the broader area of 15 km × 6 km (Fig. 6a). It is well known that molten magma, as well as partially molten rock, will slow down the propagation speed of P-waves. Also, the delay from seismic wave propagating through the molten magma is significantly larger than that from wave propagation through partially molten rock. Thus, the lack of a significant difference in P-wave delay between seismic stations with and without S-wave shadows may simply suggest that a thin molten layer may be overlying thick partially molten rocks at the lower crust beneath the TVG of northern Taiwan (Fig. 6b). In other words, the travel-time delay through the thin molten layer was significantly smaller than that caused by the thick partially molten rocks. A similar magma reservoir model of a molten magma layer overlying a partially molten low velocity zone was observed at the East Pacific Rise^24^. Although the tectonic setting between the East Pacific Rise and the TVG are different, the generation of magma reservoir might be similar because both of them are under the extensional regime^17^.

Alternatively, the presence of many molten sills as inferred in the lower crust beneath the Toba caldera^25^ might be another plausible model explaining the lack of a significant difference in P-wave delay between seismic stations with and without S-wave shadows (Fig. 6c). Again, comparing with thick partially molten rocks, the travel-time delay caused by sills might not be significant. Based on the experimental data and numerical modeling, Annen *et al*.^26^ proposed a model of “deep crustal hot zones” for the generation of intermediate and silicic igneous rocks. In the model, the mantle derived basalt emplaced into the lower crust as a succession of sills. Then partial crystallization of basalt sills not only can generate residual H_2_O-rich melts, but also provide heat and H_2_O for partial melting of pre-existing rocks. For examples, at the higher pressure (1.2 GPa), melt fraction would be ~0.5% if one considered H_2_O is 2.5 w% at temperature of 1100 °C (Fig. 5 in Annen *et al*. 2006). Under the same condition, the melt fraction would increase to ~0.56% at the lower pressure (1.0 GPa). However, there is not enough information to distinguish which model might be the most acceptable at this moment. Regardless of the exact model, both S-wave shadows and P-wave delays suggest that a magma reservoir may exist beneath the Taipei metropolis due to the ascending melt from the asthenospheric mantle^17^.

The delay of approximately 0.4 sec in P-wave arrivals provides further information for the estimation of the possible thickness of a low-velocity zone either in the lower crust or around the Moho depth. Assuming a P-wave velocity of 6.5 km/s in the lower crust, the thickness of the low velocity zone would be about 3.9 km, 6.1 km, and 10.4 km for 60%, 70%, and 80% of the velocity of the lower crust, respectively. Based on the model calculation of seismic velocity from petrofabrics and average shape of the melt phase^24^, the low-velocity zones of 60%, 70%, and 80% velocity of the lower crust are roughly associated with melt fractions of approximately 34%, 23%, and 14% in gabbro, which almost has the same geological composition as basalt in the lower crust. In other words, the thickness of the low-velocity zone caused by 14% partially molten rocks is approximately 10.4 km, but the thickness of the low-velocity caused by 34% partially molten rocks will be only approximately 3.9 km. With a larger fraction of partial melting, the layer will be thinner. Considering 34% partial melting within the magma reservoir, its size could be approximately 350 km^3^ (3.9 km × 15 km × 6 km). If 14% partial melting was estimated, the reservoir’s size might be up to 936 km^3^ (10.4 km × 15 km × 6 km).

In summary, a deep magma reservoir has been identified beneath the Taipei metropolis of Taiwan based on both S-wave shadows and P-wave delays. The magma reservoir is probably composed of either a thin magma layer overlaying a thick partially molten zone or many molten sills within the partially molten rocks. The size estimate of the magma reservoir is dependent on the fraction of partial melting within it. Considering 34% partial melting within the magma reservoir, its size could be approximately 350 km^3^. Alternatively, the size of magma reservoir may be up to 936 km^3^ if the partial melting is only 14%. Although the exact depth is not yet well constrained, a detailed image of the magma reservoir will be obtained in the next four years (Fig. S4). A new project is planned to deploy 120 broadband seismic stations with a spacing of ~5 km to create a grid covering northern Taiwan, 45 ocean-bottom-seismometers with a spacing of ~10 km offshore from northern Taiwan, and 500 geophones (3-components) with a spacing of ~500 m at the TVG in 2017–2020.

## Additional Information

**How to cite this article**: Lin, C.-H. Evidence for a magma reservoir beneath the Taipei metropolis of Taiwan from both S-wave shadows and P-wave delays. *Sci. Rep.* **6**, 39500; doi: 10.1038/srep39500 (2016).

**Publisher's note:** Springer Nature remains neutral with regard to jurisdictional claims in published maps and institutional affiliations.

## Supplementary Material

Supplementary Information
