# Supplementary material for: Evidence for a magma reservoir beneath the Taipei metropolis of Taiwan from both S-wave shadows and P-wave delays
Source: Sci Rep. 2016 Dec 23;6:39500. doi: 10.1038/srep39500 (PMC5180088; doi:10.1038/srep39500)
Supplement: Supplementary Information [file srep39500-s1.pdf]

Evidence for a magma reservoir beneath the Taipei metropolis of Taiwan from both S-wave shadows and P-wave delays

Cheng-Horng Lin[1,2,3,4,*]

1. Institute of Earth Sciences, Academia Sinica, Taipei, Taiwan
2. Taiwan Volcano Observatory at Tatun, Taipei, Taiwan
3. Dept. of Geosciences, National Taiwan University, Taipei, Taiwan
4. National Center for Research on Earthquake Engineering, National Applied Research Laboratories, Taipei, Taiwan

(*) Corresponding author:

Cheng-Horng Lin

P O Box 1-55, Nankang, Taipei, Taiwan

Email: lin@earth.sinica.edu.tw

Tel: 886-2-27839910 ext. 521

Fax: 886-2-27839195

Addition Information:

The author has no competing financial interests.

Submitted to **Scientific Reports**
On July 4, 2016
The 1st revised on September 29, 2016
The 2nd revised on November 17, 2016

SUPPLEMENTARY MATERIALS:

Table S1 Earthquake parameters used in this study.

No.	Time (UTC) yr/mon/dy	hr:min:sec	Longitude (°E)	Latitude (°N)	Depth (Km)	Mag. (M_L)
1	2015/11/28	00:52:23	122.261	25.188	219	5.15
2	2015/12/06	05:06:17	122.393	24.755	108	4.32
3	2015/04/13	01:30:28	122.399	25.286	228	4.38
4	2015/12/15	00:26:27	122.314	25.234	217	4.11
5	2015/12/16	10:30:57	122.130	25.223	218	4.28

(Provided by Central Weather Bureau, Taiwan)

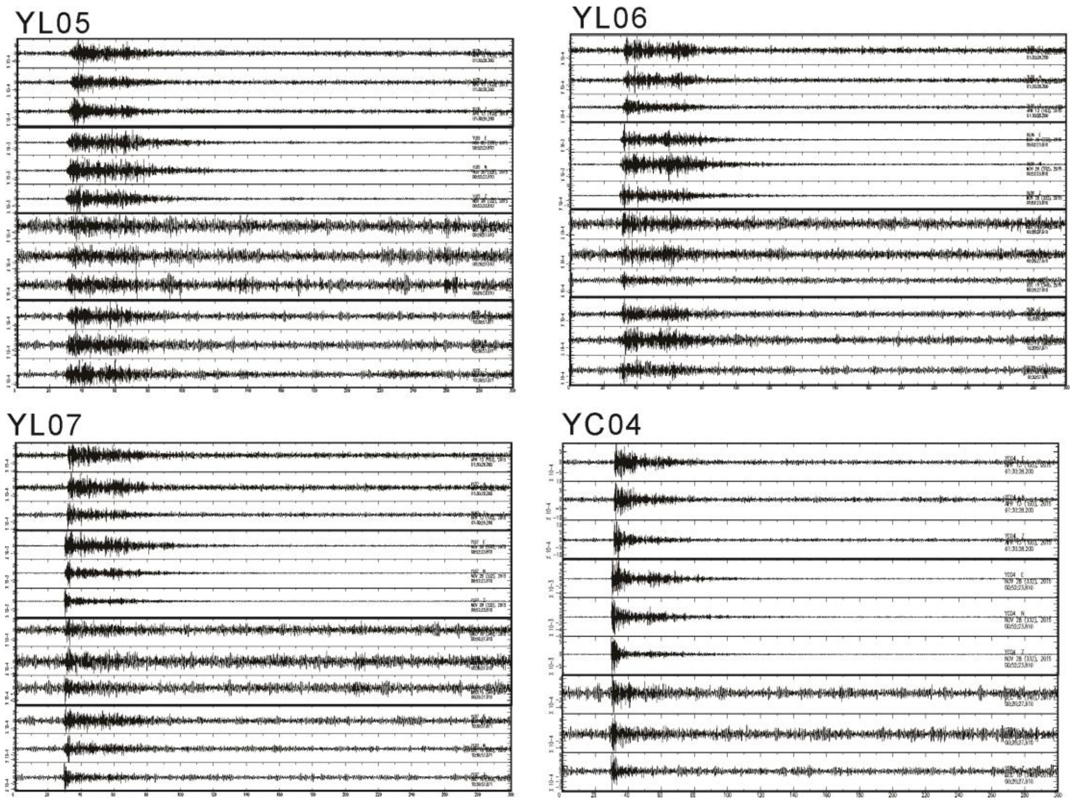

Fig. S1 Three-component seismograms generated by 4 deep earthquakes (No. 1, 3, 4 and 5 in Table S1) and recorded at 4 seismic stations (YL05, YL06, YL07 and YC04) for showing the S-wave shadow (attenuated). This figure was plotted with SAC (version 101.5; URL: ds.iris.edu).

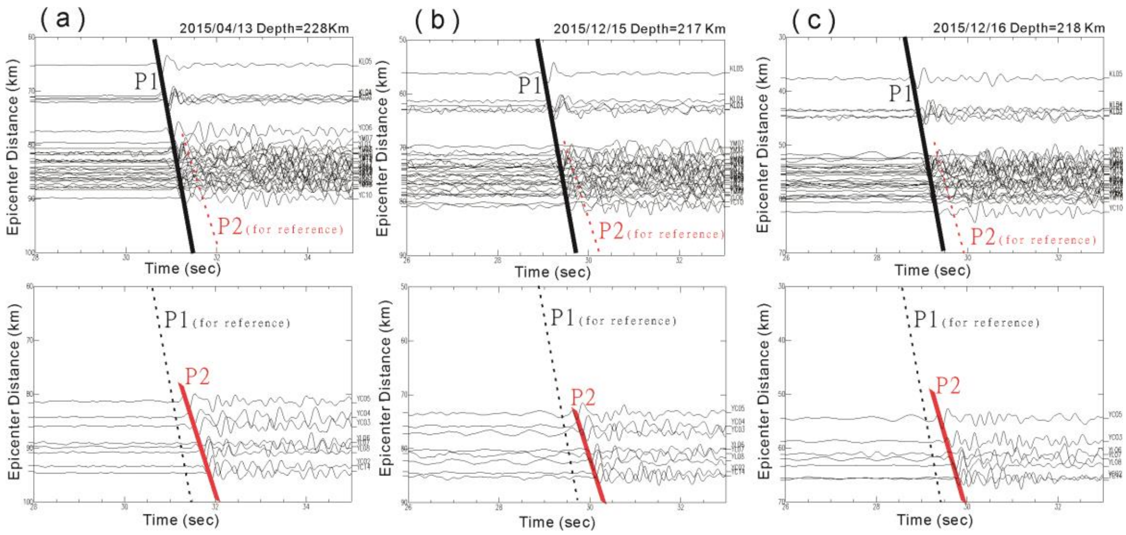

Fig. S2 Vertical seismograms plotted with the epicenter distance for showing the first P waves (P1) in the upper panel and delay P waves (p2) in the lower panel generated by deep earthquakes on (a) April 13, 2015, (b) December 15, 2015 and (c) December 16, 2015. The thick lines are fitted by the arrivals, while the dashed lines are for reference. This figure was plotted with SAC (version 101.5; URL: ds.iris.edu).

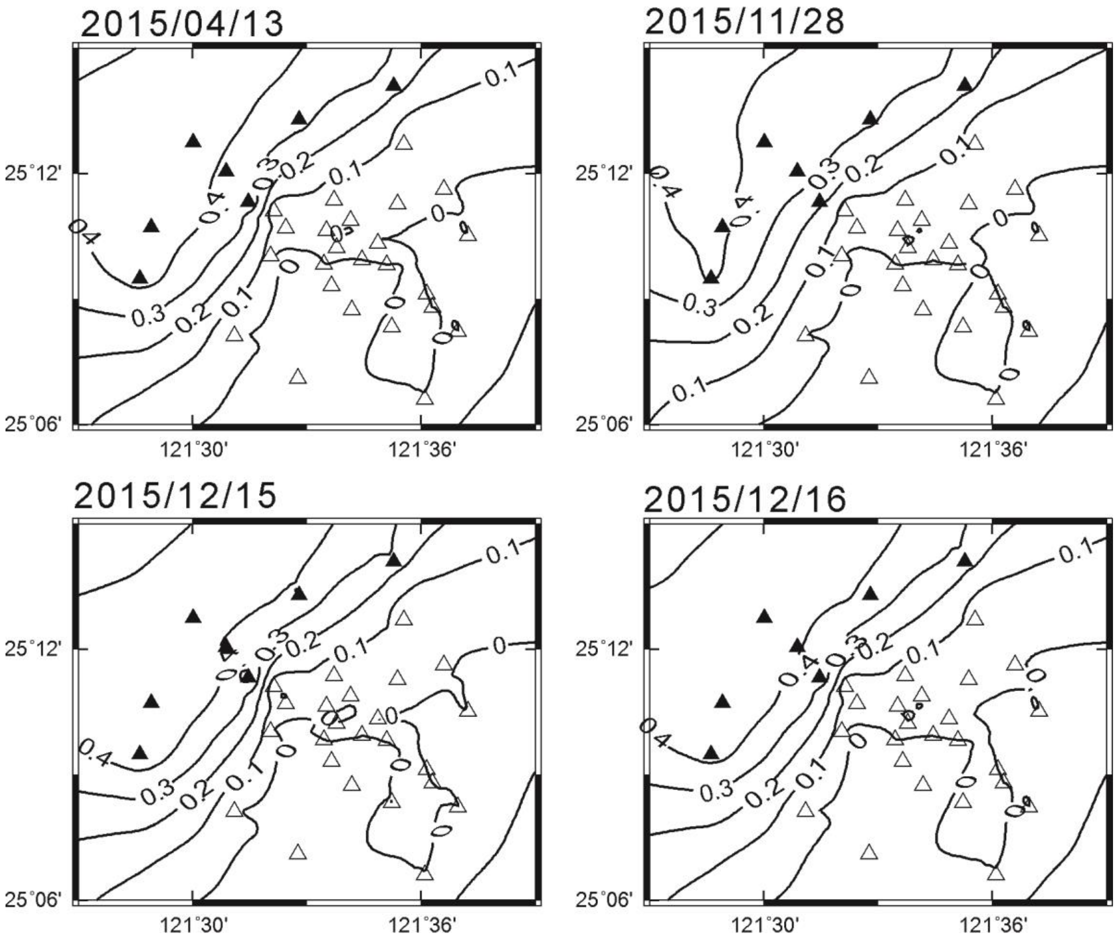

Fig. S3 The contours (s) for showing estimated P-wave delays (P2) relative to the normal arrivals (P1) in Fig. 4 and Fig. S1 of 4 deep earthquakes (No. 1, 3-5 in Table S1). The locations of seismic stations with normal P-wave arrivals (open triangles) and delays (black triangles). This figure was plotted by GMT-system-4.5.2 (gmt.soest.hawaii.edu).

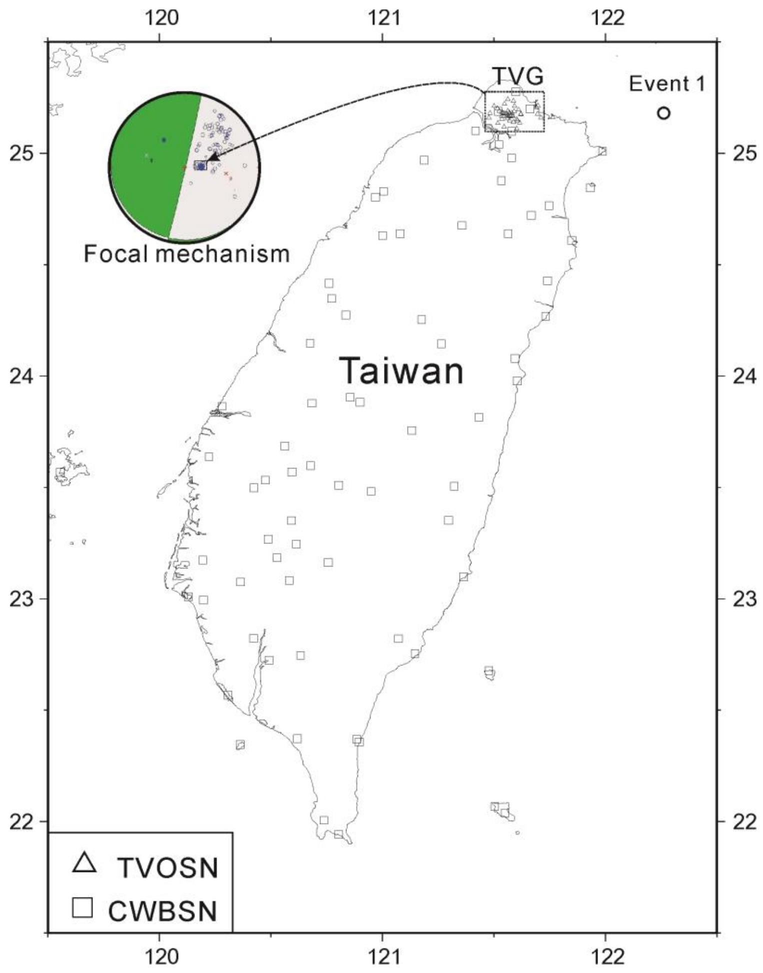

Fig. S4 Locations of both seismic networks (TVOSN and CWBSN) in the Taiwan area and the focal mechanism determined by the 1st-motion P-wave from the first representative earthquake occurred on Nov. 28, 2015. This figure was plotted by GMT-system-4.5.2 (gmt.soest.hawaii.edu).

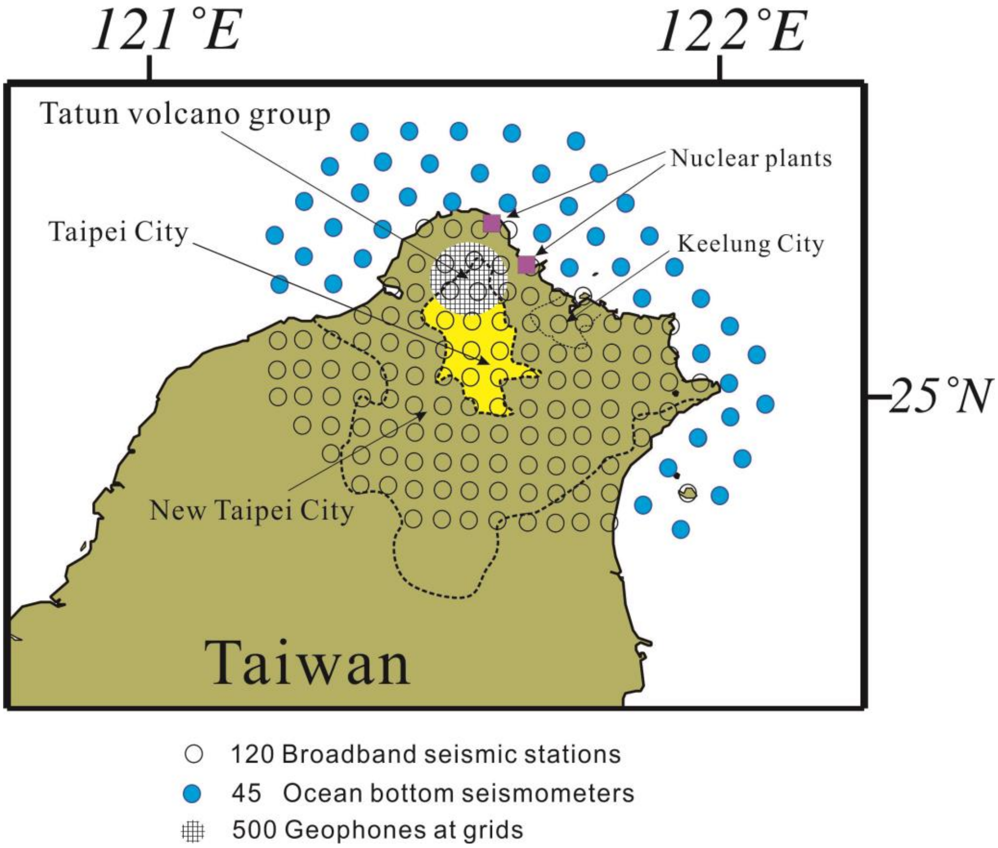

Fig. S5 Three dense seismic arrays will be deployed in northern Taiwan in 2017-2020. They include 120 broadband seismic stations (open circles) with inland spacing of ~5 km, 45 ocean bottom seismometers (blue circles) with offshore spacing of ~10 km, and 500 geophones with spacing of ~500 m at the Tatun volcano group (the grid area). This figure was plotted by GMT-system-4.5.2 (gmt.soest.hawaii.edu), and data for plotting the maps were provided by NGIS (ngis.nat.gov.tw/3_3_1.aspx).
